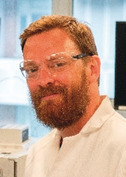# Synthetic Organic Design for Solar Fuel Systems

**DOI:** 10.1002/anie.202006013

**Published:** 2020-07-29

**Authors:** Julien Warnan, Erwin Reisner

**Affiliations:** ^1^ Department of Chemistry University of Cambridge Lensfield Road Cambridge CB2 1EW UK; ^2^ Department Chemie Technische Universität München Lichtenbergstraße 4 85747 Garching Germany

**Keywords:** electrocatalysis, molecular electronics, photocatalysis, sustainable chemistry, synthesis design

## Abstract

From the understanding of biological processes and metalloenzymes to the development of inorganic catalysts, electro‐ and photocatalytic systems for fuel generation have evolved considerably during the last decades. Recently, organic and hybrid organic systems have emerged to challenge the classical inorganic structures through their enormous chemical diversity and modularity that led earlier to their success in organic (opto)electronics. This Minireview describes recent advances in the design of synthetic organic architectures and promising strategies toward (solar) fuel synthesis, highlighting progress on materials from organic ligands and chromophores to conjugated polymers and covalent organic frameworks.

## Graphical abstract

**Organic and hybrid systems** have emerged to challenge the classical inorganic structures through their enormous chemical diversity and modularity. Recent advances in the design of synthetic architectures and promising strategies toward (solar) fuel synthesis are discussed, highlighting progress on materials from dyes and catalysts to polymers and covalent organic frameworks.

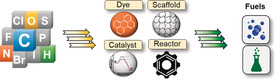

## 1 Introduction

Since the demonstration of photoelectrochemical water splitting using the semiconductor (SC) TiO_2_ and the H_2_ evolution catalyst (HEC) platinum, inorganic materials have dominated the field in both number and efficiency.^1^ They still undergo fast development, but often lack understanding at an atomic level, which limits the flexibility and fine‐tuning capabilities needed to rationally improve (photo)catalytic performance.

Biology, through photosynthesis and fuel‐making enzymes even in non‐photosynthetic organisms, provides blueprints for the design of dyes and catalysts with outstanding performance such as the light‐harvesting complexes, H_2_‐evolving hydrogenase (H_2_ase) and the CO_2_ reductases.^2^ The activity of these biocatalysts relies on the choreography of evolutionarily‐developed design principles, including: active sites with an optimized primary and outer coordination sphere to stabilize reaction intermediates, efficient energy and electron transfers (ETs), well‐aligned electroactive ligands and electron relays, as well as substrate and product channels.

Although some of these concepts are being implemented toward artificial photosynthesis and (photo)catalysis, especially in the design of molecular electrocatalysts, most reports focus on inorganic systems. In addition to coordination complex catalysts and natural archetypes, (semi‐)organic (hybrid) materials have emerged in the field of catalysis.^3^ The modularity and amenability displayed by these materials offer a fertile ground for integration in catalytic schemes, which have led to rapid developments in organic photo‐ and electrochemistry.^4^

Here, we summarize the progress toward developing electro‐ and photo‐catalysis systems for fuel synthesis enabled by organic design, organized according to molecular and polymeric concepts. Although the synthetic strategies described in this mini‐review focus on the fuel forming, reductive half‐reaction, analogous design can also be employed in water oxidation catalysis with oxidatively robust organic architectures.

## 2 Discrete Molecular Systems

### 2.1 Ligand Design for Catalysts

**Figure 1:**
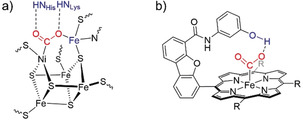
(a) [NiFe] carbon monoxide dehydrogenase active site^9^ and (b) synthetic Fe‐porphyrin^10^ with bound CO_2_ (in red) stabilized by outer coordination sphere interactions (in blue).

The [NiFe]‐ or [FeFe]‐active site in H_2_ase and the O_2_‐evolving [CaMn_4_]‐cluster in Photosystem II (PSII) inspired the development of early structural biomimetics as *molecular catalysts*.^5^ Catalysts were initially designed to mimic the first coordination sphere of the enzyme active site, which led to Fe_2_S_2_‐type HECs delivering modest performances,5f, 6 and having the propensity to decompose into active particles.^7^ Tuning the ligands’ substituents to affect the electronic density and electrochemical properties was also extensively investigated.5e, 5f, 6c A more recent approach is to innovate modulation of the second and outer coordination spheres of the metal center with organic residues in an attempt to replicate the multifunctionality found in enzymes (Figure 1).6c, 8

**Figure 2:**
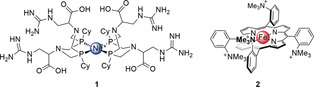
Catalysts with engineered ligands to stabilize reaction intermediates via secondary coordination sphere interactions. The functional groups in **1** are drawn as neutral for simplicity, but this may not reflect the real protonation state.

A class of Ni‐containing HECs containing a P_2_N_2_ ligand (Dubois catalyst) displays high activities due to pendant basic tertiary amines that promote proton transfer.^11^ HEC **1** with further arginine (Arg) residues displayed reversible H_2_ production/oxidation in acidic aqueous solutions (Figure 2).8c The high turnover frequency (TOF) of 300 s^−1^ was attributed to Arg–Arg interactions that aid positioning of the pendant‐amine groups in close proximity to the Ni center.

Tuning porphyrin ligands to improve the performance of a CO_2_ reduction catalyst (CRC) was demonstrated with Fe‐tetraphenylporphyrins (TPPs).8d, 10, 12 Fe‐TPPs display a catalytic onset potential (*E*_cat_) of −1.40 V vs. standard hydrogen electrode (SHE) in *N*,*N*‐dimethylformamide (DMF).^12^ The catalytic performance was optimized by stabilization of the initial Fe^0^‐CO_2_ adduct upon addition of positively charged *N*,*N*,*N*‐trimethylanilinium groups in the *ortho* position of the four phenyl groups in **2** (Figure 2). This modification resulted in a more anodic *E*_cat_ of −0.95 V vs. SHE with a 3‐fold greater catalytic current than the corresponding “*para*” catalyst, while also delivering high Faradaic efficiencies (FEs) toward CO with limited degradation over 84 h of electrolysis in DMF containing phenol and H_2_O.8d The catalysis‐enhancing effect was attributed to Coulombic interactions of the positively charged moieties with the carboxylate borne from the Fe^0^‐CO_2_ adduct.

Other concepts to improve performance have been reviewed elsewhere and include (non‐exhaustively): modulating steric hindrance around the metal core,^13^ isolating catalysts on surfaces via anchoring groups,^14^ providing Brønsted acid groups to deliver proton relays,8b H‐bonded and multimetallic systems for electrostatic stabilization of CO_2_‐bonded intermediates,^15^ and tailored CO_2_‐fitting clefts.^10^

Importantly, as water oxidation represents a scalable and readily available source of electrons for (solar) fuels production, many molecular catalysts for the oxygen evolution reaction have been developed with ligand design toward modulating the catalyst's outer coordination sphere also representing an active area of research.5a, 16 In particular, Ru coordination complexes currently display benchmark performance and more in‐depth reviews on this topic can be found elsewhere.^17^

### 2.2 Dyes

**Figure 3:**
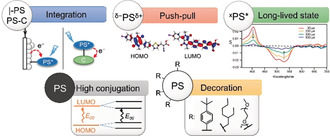
Examples of properties implemented in chromophores for optimizing photocatalytic performance (C=catalyst). Adapted with permission from ref. 19 and 20. Copyright (2011 and 2018) Elsevier and American Chemical Society.

Photosensitizers (PSs) can harvest light to drive a suitable electrocatalyst for solar fuel synthesis. Common homogeneous photocatalytic systems employ commercial dyes, e.g., Ir and Ru complexes, that are regenerated by a sacrificial electron donor (SED). Purely organic PSs, now ubiquitous in dye‐sensitized solar cells (DSCs), have been much less investigated for the demanding requirements of artificial photosynthesis such as aqueous conditions and endergonic multi‐electron processes.^18^ Common limitations include their lack of solubility in aqueous media, modest stability and short‐lived excited states that impede diffusional ET. Building on chromophoric units, drawbacks can be overcome by molecular engineering to yield suitable PSs (Figure 3).

**Figure 4:**
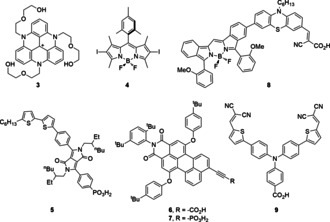
Organic PSs used in photocatalytic fuel synthesis.

For instance, triazatriangulenium **3** (Figure 4) displays intense visible‐light absorption (*λ*_max_=530 nm; *ϵ*=8800 m ^−1^ cm^−1^) with a relatively long excited‐state lifetime of its singlet state (14 ns at pH 4.5).^20^ This allows in solution for a diffusion‐controlled reductive quenching by ascorbic acid (AA) to generate the organic radical **3** ^.−^ with excellent stability due to the planar scaffold incorporating three electron‐donating nitrogen atoms for delocalization of the radical. As a result, efficient ET to a molecular Co HEC was observed, delivering a PS‐based turnover number (TON_PS_) of ≈100 (limited by the HEC′s stability).

An alternative PS‐design strategy to improve intermolecular ET is the promotion of intersystem crossing toward a long‐lived triplet state. Halogenation of a borondipyrromethene (bodipy) PS yields diiodide‐bearing **4** (Figure 4) that has a considerably shorter fluorescence lifetime than the corresponding iodine‐free PS (0.13 vs. 6.0 ns, respectively), indicating fast intersystem crossing.^21^ PS **4** reached a TON_PS_ of 600 when employed at low concentrations with a molecular Co HEC and triethanolamine (TEOA) as a SED in acetonitrile under Xe/Hg lamp irradiation.

**Figure 5:**
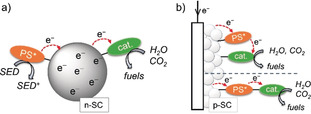
Schematic representation of (a) colloidal DSP and (b) DSPC schemes, with a PS and catalyst (cat.) or a PS‐cat. dyad co‐anchored onto a SC electrode.

Organic dyes have also been involved in colloidal dye‐sensitized SC photocatalysis (DSP) systems toward H_2_ evolution and CO_2_ reduction (Figure 5 a).^22^ Building upon DSC principles, DSP systems are assembled through attachment of an anchor‐bearing PS to the surface of a SC particle, together with a co‐attached catalyst.18a, 22a The SC delivers dual functionality as it provides a scaffold for immobilization to enable fast charge separation and allows accumulation of multiple long‐lived charges in the SC to drive catalysis. The regeneration of the photoionized PS generally relies on a SED.

A series of diketopyrrolopyrrole (DPP) dyes with modulated energetic and non‐energetic parameters (e.g., steric hindrance, position and nature of the solubilizing side chains) were studied in DSP systems.^23^ When attached to platinized TiO_2_ and placed under simulated sunlight, PS **5** (Figure 4) delivered a performance (TON_PS_ in **5**|TiO_2_|Pt ≈2700) superior even to the corresponding phosphonic acid‐bearing Ru trisbipyridine‐based assembly. The performance of the PS in DSP systems was shown to ultimately depend on the orthogonal adequacy of PS design and external parameters (pH, SED, chemical catalyst and mechanistic details).

A series of hydrophobic perylene monoimide (PMI) dyes were functionalized with five different anchoring groups: carboxylic acid, phosphonic acid, acetylacetone, pyridine‐2,6‐dicarboxylic acid and hydroxyquinoline. These PSs were investigated in DSP using platinized TiO_2_ nanoparticles for H_2_ evolution in water.^24^ The CO_2_H‐bearing PS **6** (Figure 4) delivered the best performance in acidic and neutral pH with a stability beyond 3 days and a TON_PS_ of ≈11 000. The activity decreased at higher pH due to desorption of the PS from the SC surface. In contrast, albeit yielding modest activity, the phosphonic acid‐bearing PS **7** (Figure 4) enables good stability due to the anchoring group's better resistance to hydrolysis. These results highlight an ongoing challenge in DSP, where electron injection‐promoting properties are not yet readily compatible with robust anchoring.

The tunable electronic properties of organic chromophores allow their use for the absorption and conversion of low‐energy photons. PS **8** (Figure 4) shows the extended conjugation of a bodipy to a phenothiazine, with its donor‐π conjugated linker–acceptor (d–π–A) organization leading to a strong panchromatic absorption up to 700 nm with *λ*_max_=638 nm (*ϵ*=123,000 m ^−1^ cm^−1^). Attachment of **8** on platinized hierarchical porous TiO_2_ resulted in a DSP system toward H_2_ evolution, with a TON_PS_ of 11 100 after 10 h of irradiation (λ>400 nm, 100 mW cm^−2^) in the presence of AA as SED. An apparent quantum yield (QY) of 1.0 % at 750 nm was recorded.^25^

Overcoming the parasitic, fast electron recombination in PS‐catalyst systems as well as suppressing the need for a SED are current challenges in colloidal and homogeneous schemes. Anchoring a dye and catalyst onto a p‐type SC (p‐SC) electrode to produce a dye‐sensitized photocathode (DSPC, Figure 5 b) is a promising strategy inspired by p‐type DSCs to address these limitations.3c, 26 PS **9** (Figure 4) was co‐anchored to a nanostructured NiO substrate together with a cobaloxime HEC.^27^ The push‐pull design in **9** locates the dye's highest occupied molecular orbital close to the NiO surface, thereby promoting hole injection and intermolecular ET to the HEC upon photoexcitation. A fully assembled tandem photoelectrochemical cell with a dye‐sensitized photoanode demonstrated overall water splitting.^27^ Incident photon to current conversion efficiency analysis identified the photocathode as the overall system's bottleneck, ascribed to the limitations of NiO (e.g., short hole diffusion length). Alternative p‐SCs such as CuCrO_2_ (combined with PSs **5** and **7**) and LaFeO_3_ have recently been proposed as a potential replacement for NiO.^28^

### 2.3 Molecular Electro‐ and Photo‐catalysis

Photocatalysts require structures with high molar absorption and a reactive catalytic center.^29^ The difficulty of combining these two properties in one discrete molecule led to the development of architectures that covalently or supramolecularly bind a dye to an electrocatalyst,^30^ where the photoexcited PS triggers intramolecular ET to the catalyst site. Such dyads often rely on a precious metal‐based PS, but organic chromophores are emerging.

**Figure 6:**
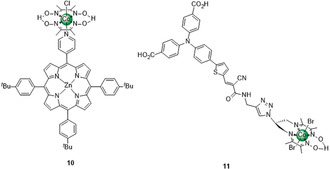
Photocatalysts for H_2_ production based on transition metal‐free PSs.

Cobaloxime‐based dyads are usually assembled from the HEC unit bound axially to a pyridine‐functionalized PS. An example is the Zn–porphyrin–cobaloxime dyad **10** (Figure 6), which photogenerated a TON${}_{H{}_{2}}$ of 22 after 5 h irradiation (λ>400 nm) in H_2_O:tetrahydrofuran (THF) (1:4) with triethylamine (TEA) as SED.30a No H_2_ was detected when using Zn‐porphyrin not bound to the cobaloxime.

A photocatalyst composed of a cobalt diimine‐dioxime HEC linked to the carboxylate anchor‐bearing PS **11** (Figure 6) was used as a dyad in a DSPC (PS‐cat., Figure 5 b).30d The PS's push–pull design locates the lowest unoccupied molecular orbital close to the HEC and thereby promotes its intramolecular reduction upon irradiation. As a result, a NiO|**11** DSPC displayed an early photocurrent onset potential of +0.61 V vs. SHE, and chronoamperometry at −0.18 V vs. SHE in pH 5.5 electrolyte solution under simulated solar irradiation resulted in a FE${}_{H{}_{2}}$ of 8–10 %.

**Figure 7:**
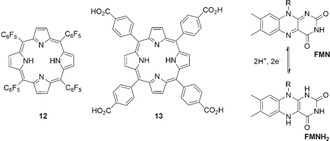
Metal‐free compounds for electro‐ and photo‐driven fuel production.

**Figure 8:**
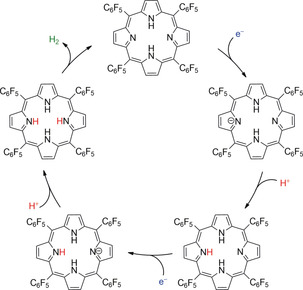
Proposed H_2_ evolution mechanism for **12**.^32^

Another challenge for metal‐free molecular systems is delivering fuel production activity.^31^ A free‐base porphyrin (P2H) bearing four electron‐withdrawing *meso*‐tetra(pentafluorophenyl) groups (**12**, Figure 7) achieved H_2_ production.^32^ Cyclic voltammetry conducted in THF featured two reversible 1‐e^−^ reductions at *E*_1/2_=−1.14 and −1.54 V vs. ferrocene/ferrocenium (Fc/Fc^+^). Upon addition of tosic acid, the first reduction wave remained unchanged, while an enhanced wave appears at −1.31 V vs. Fc/Fc^+^, indicating that protonation of the porphyrin occurs in the cavity after the first reduction (Figure 8). H_2_ evolution was observed during bulk chronoamperometric experiments at −1.7 V vs. Fc/Fc^+^, with 90 % FE after 40 min and a calculated TON <1.

Another P2H (**13**, Figure 7) is capable of photo‐catalyzing the production of H_2_O_2_ from O_2_ when immobilized on NiO.^33^ H_2_O_2_ has potential as a liquid fuel with an energy density comparable to that of compressed H_2_. Bulk electrolysis of NiO|**13** at ≈+0.20 V vs. SHE (pH 6) under 623 nm LED irradiation for 24 h resulted in near‐unity FE and a TON >12,000. The production of H_2_O_2_ was attributed to the light‐driven reduction of O_2_ by **13** into the superoxide radical anion O_2_^.−^, which further disproportionates into H_2_O_2_.

Flavins are organic electro‐ and photo‐catalysts studied toward chemical oxidation chemistry that commonly use O_2_ as the final electron acceptor in order to produce H_2_O_2_.^34^ Similar to quinone, flavin‐derivatives exist in three redox states: neutral (flavin‐quinone **FMN**, Figure 7), 1 e^−^‐reduced, and 2 e^−^‐reduced (flavin‐hydroquinone, **FMNH_2_**). Upon irradiation with blue light, the **FMN** chromophores form a singlet excited state, a potent oxidant with *E*(FMN*/FMN^.−^)=+1.53 V vs. SHE. Rapid intersystem crossing (7.8 ns in water) also produces a triplet state that triggers ET. **FMNH_2_** is often ultimately oxidized to **FMN** in presence of O_2_. Alternative mechanisms could also be occurring such as FMN* converting ^3^O_2_ into a reactive ^1^O_2_ species.34c

## 3 Polymeric Systems

Whilst Nafion^®^ remains the most commonly used polymer, alternatives are quickly being developed for solar fuel applications. Electropolymerization was employed early to surface anchor molecular catalysts via pyrrole, vinyl and methacrylate groups for applications in water oxidation, proton and CO_2_ reduction.^35^ Recent reports have considerably expanded the scope of bespoke polymers toward solar fuel production with applications ranging from scaffolds and catalysts to PSs.

### 3.1 Scaffolds for Electrocatalysts

The active sites of enzymes are embedded into polypeptide scaffolds. Synthetic polymeric matrices can similarly integrate HECs or CRCs to provide better stability, and functionalities to stabilize catalytic intermediates or allow for surface anchoring.^36^

**Figure 9:**
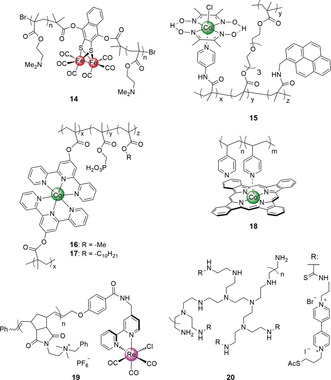
Scaffolding polymers for electrocatalytic systems.

An amine‐containing metallopolymer **14** (Figure 9) derived from 2‐(dimethylamino)ethylmethacrylate and a Fe_2_S_2_‐type HEC operates in pH neutral aqueous solution with high current densities, a TON of 2.6×10^4^, an operational lifetime of 6 days and even retained activity under aerobic conditions. The high performance is possibly due to the protonation of amine side‐chains that facilitates proton transport to the Fe‐catalyst while shielding it from O_2_ reduction products.36d

A cobaloxime HEC was integrated into a cross‐linked copolymer via a pyridine ligand that also contained pyrene and ethylene glycol groups (**15**, Figure 9). Interfacing this structure with carbon nanotubes resulted in a standalone electrode achieving higher TONs and stability compared to an electrode with the immobilized monomeric cobaloxime HEC. The improved performance was attributed to entrapment of the otherwise labile Co HEC in the polymeric matrix and improved proton transport from the ethylene glycol moieties. This work highlights the potential benefits by considering the choice of co‐monomers, independent of the HEC unit itself.36a

**Figure 10:**
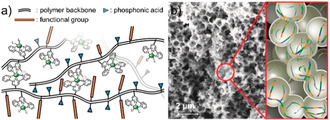
(a) Schematic representation of **16** and **17**. (b) Scanning electron microscopy image of inverse opal TiO_2_ and schematic of polymer chains embedded in the porous scaffold. Reprinted with permission from ref. 36e. Copyright (2019) John Wiley and Sons.

A similar copolymeric approach was applied toward CO‐selective CO_2_ reduction using a Co bis(terpyridine) CRC. Two coordination copolymers were prepared comprising the CRC, phosphonic acid anchoring groups, and either a methyl or decyl moiety to tune hydrophobicity surrounding the catalyst core in **16** and **17**, respectively (Figure 9 and 10 a). After integration of the copolymers into bespoke inverse opal TiO_2_ electrodes (pore Ø=750 nm, Figure 10 b), electrolysis demonstrated higher selectivity toward CO vs. H_2_ for the more hydrophobic, decyl‐based copolymer, highlighting the possibilities for improved product selectivity offered by tuning the catalyst's outer sphere environment.36e

Poly‐4‐vinylpyridine coordinated to a Co phthalocyanine (CoPc) in sub‐stoichiometric conditions resulted in polymer **18** (Figure 9) that was interfaced with graphite electrodes. A better electrolysis performance for CO_2_ reduction was achieved compared to the corresponding pyridine‐coordinated, molecular CoPc, which was attributed to outer sphere effects of uncoordinated pyridines.36b, 36g

A series of polymeric frameworks with positively charged ammonium salts, phenyl, or negatively charged trifluoroborate groups was designed to alter the catalytic activity of a covalently bound Re CRC.36c Electrochemical studies in organic solvent showed that the quaternary ammonium‐containing polymers **19** (Figure 9) have a significantly lower *E*_cat_ toward CO evolution (≈300 mV) compared to the free, molecular catalyst. In contrast, the trifluoroborate polymers displayed a negative shift in potential and catalytic activity was not observed. This illustrates how a charged polymeric framework can influence the catalytically active species without changing the primary coordination environment around the reactive center.

Using a semi‐biological approach,^37^ a multifunctional polymer was employed as a hydrogel to provide stabilization and entrapment to an O_2_‐sensitive [NiFe]‐H_2_ase toward biofuel cell applications.37a, 37d The polyamine‐based polymer **20** (Figure 9) was synthesized from a branched poly(iminoethylene) functionalized with electroactive methyl viologen (MV) units.37a The MV units in **20** act as an electron relay for enzymatic catalysis and reduce O_2_ to protect the H_2_ase. A few micrometer‐thick polymer film is sufficient to protect the H_2_ase without compromising the current generation efficiency.37d

A phenothiazine‐based polymer was employed to wire PSII to a porous electrode for photoelectrocatalytic water oxidation.37b Integration of PSII via a polymer‐matrix to a dye‐sensitized photoanode (using PS **5**) enabled unassisted overall water splitting with a cathode containing H_2_ase.37c

### 3.2 Polymeric Dyes and Photocatalysts

Light harvesting and charge conducting SCs are ubiquitous in lightweight optoelectronics applications and typically produced from conjugated polymers (CPs). CPs can be produced under mild conditions with molecularly tunable optoelectronic and physicochemical properties.^38^ Recently, they have also emerged as promising materials for photocatalytic fuel production delivering high catalytic activities, often when combined with added or residual Pd/Pt nanoparticles.3d In some cases, thorough metal removal and purification has been shown to eliminate activity, demonstrating that residual Pd—even at ppm‐level (e.g. from cross‐coupling reactions)—plays a significant role in the H_2_ evolution abilities of some polymers.^39^

**Figure 11:**
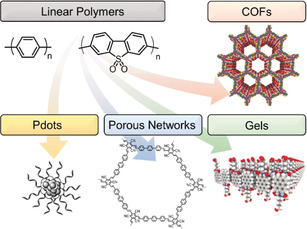
Evolution from linear polymeric aggregates to controlled 2D/3D polymeric architectures. Adapted with permission from ref. 43. Copyright (2016, 2014 and 2018) John Wiley and Sons and Springer Nature.

CPs have evolved from linear polymers to non‐crystalline microporous polymer networks, carbon nitrides and carbon dots, polymer dots (Pdots) and covalent organic frameworks (COFs) (Figure 11).^40^ Carbon nitride derivatives (and other triazine‐based systems) and carbon dots (along with other carbon nanoparticles) are attracting much attention,^41^ and they can be fabricated from purely organic precursors (e.g., melamine, urea, cyanamide, citric acid and aspartic acid) via pyrolysis and solvothermal procedures at relatively high temperatures.3b, 41a, 41c, 42 Modulation of their photocatalytic activity generally involves solid‐state approaches to introduce morphological alteration, doping and composite construction.3b, 41b Given that the synthetic procedures, tuning approaches and properties of these materials differ significantly from the molecularly‐defined polymeric materials described in this section, we will not examine them further. Reviews on carbon nitrides and carbon dots can be found elsewhere.3b, 42a Hereafter, we focus on polymer engineering strategies to enhance activities, including improved light‐harvesting properties, porosity and crystallinity.

**Figure 12:**
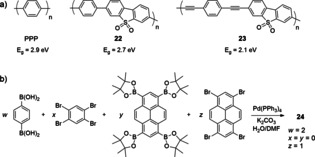
Tuning the band gap in (a) linear and (b) 3D microporous CPs.

CPs offer the possibility of fine‐tuning SC properties such as the energy levels and resulting optical band gap (E_g_), through the selection of monomeric building blocks and via modular polymerization strategies. An early report showed that poly(*p*‐phenylene) (PPP, Figure 12 a) acts as a photocatalyst for H_2_ evolution, despite showing low activity and requiring UV irradiation. The E_g_ could be reduced from 2.9 to 2.7 eV (for PPP and **21**, respectively) with the introduction of a dibenzo[*b*,*d*]thiophene sulfone moiety and then further to 2.1 eV upon introduction of planarizing ethynyl groups in **22** (Figure 12 a).40a, 44 The smaller E_g_ combined with accelerated charge separation allowed for an increase in the photocatalytic rate from 1492 to 6023 μmol${}_{H{}_{2}}$ h^−1^ g^−1^ in the presence of a SED.

This strategy was also employed in 3D microporous CPs, where high surface area polymers were prepared by adjusting the ratio of four monomers (Figure 12 b).^45^ The corresponding E_g_ thereby gradually decreased from 2.95 to 1.94 eV following an increase in pyrene content.^46^ In the absence of an externally added metal catalyst, the polymers showed a gradually enhanced photoactivity for H_2_ evolution with an optimal E_g_ of 2.33 eV for **23**. Polymer **23**, obtained with a ratio of 2:1 for 1,4‐benzene diboronic acid:1,3,6,8‐tetrabromopyrene, exhibited the highest activity of 174 μmol${}_{H{}_{2}}$ h^−1^ g^−1^ (and a mid‐range surface area compared to polymers of other ratios). Further reduction in E_g_ of the polymers led to a lower rate, which was ascribed to increased nonradiative deactivation in the pyrene‐rich polymers.

The high hydrophobicity of CPs commonly results in their aggregation in aqueous solution into a bulk material composed of micrometer‐size particles with low surface area (i.e., water‐polymer interface) and extended travelling distance for charge carriers. To overcome these issues, porous systems as well as optimized precipitation and gel‐promoting methods have been developed.

**Figure 13:**
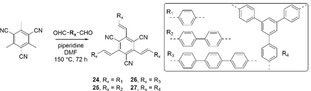
Synthesis of CPs with different porosities.^47^

The length of the π‐extended linker affects the porosity of microporous networks synthesized via condensation, as shown in polymers **24**–**27** (Figure 13).^47^ Polymer **24** with a short *para*‐phenylene spacer exhibits micropores, whereas **25** and **27** contain longer spacers and subsequently integrate micro‐ and mesopores. Polymer **26** shows a much broader pore size distribution due to the longest polyphenylene spacer in the polymer network. As a result, the Brunauer‐Emmett‐Teller (BET) surface areas were 669, 750, 564 and 834 m^2^ g^−1^ for **24**, **25**, **26** and **27**, respectively. Despite their similar E_g_, the corresponding photoactivities were 134, 598, 908 and 620 μmol${}_{H{}_{2}}$ h^−1^ g^−1^, respectively, with the highest rate for **26** and ascribed to its nanoparticular morphology, better wettability and large surface area. This highlights the possibility to influence the activity of polymers via fine‐tuning of morphological variations.

**Figure 14:**
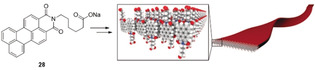
Self‐assembled chromophores resulting in a polymer hydrogel. Adapted with permission from ref. 43a. Copyright (2014) Springer Nature.

An alternative approach was developed with the charged amphiphilic PMI dye **28** (Figure 14), which can self‐assemble into ribbon‐type supramolecular polymers via hydrophobic collapse.43a Moreover, at sufficiently high concentrations, the charged supramolecular polymers produce highly hydrated 3D network hydrogels, which display a high degree of crystallinity. This leads to the PMI losing its individual excitonic character and behaving as an ensemble with photoinduced excitons spreading out over multiple PMI units within the crystalline ribbons. The **28**‐based hydrogels formed in presence of poly(diallyldimethylammonium) chloride can host a water‐soluble Dubois Ni HEC, ultimately producing a TON${}_{H{}_{2}}$ of ≈340 under irradiation in the presence of AA as SED.

**Figure 15:**
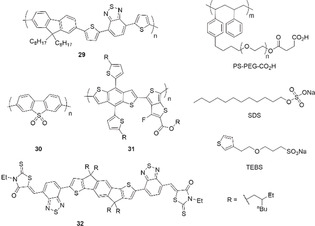
Conjugated architectures and surfactants for Pdot systems.

Pdots represent another family of organic SC assemblies used in photocatalysis with diameters from 1 to 100 nm.43b, 48 The smaller size of Pdots compared to bulk materials reduces the distance for photogenerated charges to migrate to the surface, which decreases the recombination probability. A Pdot suspension can be generated by the nano‐precipitation method using CPs and a water‐soluble polymer. For example, the synthetic polymer **29** and the matrix PS‐PEG‐CO_2_H (Figure 15) were solubilized in THF and injected into pure water under sonication to produce a suspension after solvent evaporation.^49^ Absorption up to 660 nm and a D‐A architecture in **29** allowed for an excellent H_2_ evolution rate of up to 50 mmol${}_{H{}_{2}}$ h^−1^ g^−1^ under LED irradiation in presence of AA as SED. Although **29** contains traces of Pd (0.1 wt %), theoretical calculations suggested that nitrogen atoms in the benzothiadiazole units may provide the reactive sites for the formation of H_2_.

The linear homopolymer of dibenzo[*b*,*d*]thiophene sulfone **30** produced emulsion particles when synthesized from mini‐emulsions of toluene droplets in water with water‐stabilizing sodium *n*‐dodecyl sulfate (SDS, Figure 15). The latter exhibited a high surface area of 16 m^2^ g^−1^, contained 0.4 wt % Pd, and produced H_2_ with an excellent rate of 61 mmol${}_{H{}_{2}}$ h^−1^ g^−1^ under visible light irradiation in the presence of TEA.^50^

Inspired by bulk heterojunction‐based solar cells and emerging examples of photoelectrodes,^51^ a similar approach was extended to the preparation of heterojunction nanoparticles using a blend of a donor polymer **31** and a non‐fullerene acceptor **32**, in the presence of sodium 2‐(3‐thienyl)ethyloxybutylsulfonate (TEBS) as a stabilizing agent (Figure 15).^52^ The TEBS’ affinity of its exposed aromatic units of **32** is believed to control the nanomorphology of these particles into an intermixed D/A blend. SDS, on the other hand, promotes an inefficient core–shell morphology. A 30:70 blend content of **31**:**32** is optimal for efficient exciton dissociation and formation of ≈82 nm particles. Following platinization, the photocatalyst displayed a significant H_2_ evolution rate of ≈64 mmol${}_{H{}_{2}}$ h^−1^ g^−1^ under broadband visible light illumination, and an external QY exceeding 5 % from 660 to 700 nm.

**Figure 16:**
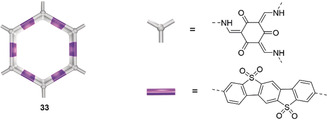
Dibenzo[*b*,*d*]thiophene sulfone‐based COF. Adapted with permission from ref. 43c. Copyright (2018) Springer Nature.

Crystallinity can improve the efficiency in conjugated systems as it favors charge transport and separation. COFs are an emerging class of 2D/3D polymers and an example of highly crystalline organic building units combined into extended covalent structures.^53^ The well‐defined pores, excellent stability and fine‐tuned physicochemical properties of COFs make them appealing candidates as PSs and catalysts for fuel production.^54^ A dibenzo[*b*,*d*]thiophene sulfone moiety can be integrated into a COF (**33**, Figure 16), which leads to a high photocatalytic performance of 10 mmol${}_{H{}_{2}}$ h^−1^ g^−1^ when used in presence of Pt and AA as SED.43c The COF allows broad visible light absorption (E_g_=1.85 eV) and relatively long excited state lifetimes (*τ*_avg_=5.56 ns) in aqueous suspensions. The high efficiency was ascribed to its good wettability and a large BET area of 1288 m^2^ g^−1^ from its 28 Å pore size diameter.

**Figure 17:**
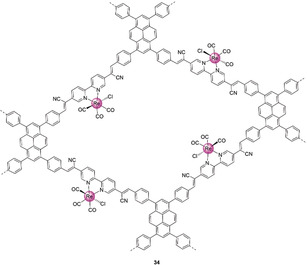
COF for photocatalytic CO_2_‐to‐CO conversion.^55^

A bipyridine‐containing COF was recently post‐synthetically modified with a Re complex to afford the photocatalyst **34** (Figure 17).^55^ The latter delivers a CO production rate of 1040 μmol_CO_ g^−1^ h^−1^ with 81 % selectivity over H_2_, across 17.5 h of illumination (TON_CO_≈19) in acetonitrile containing TEOA. Computational results support that ET occurs from the light‐absorbing COF backbone to the Re CRC upon photoexcitation. Crystallinity and porosity were key factors in the activity of such materials, as an amorphous, low porosity analogue showed almost no catalytic activity.

## 4 Conclusions

The development of synthetic organic architectures for fuel catalysis has experienced rapid progress during the past decade and we have summarized the wealth of approaches that originated from integrating organic designs. Tailor‐made organic structures have followed several approaches, ranging from small molecules to polymers acting as light harvesters, (photo)catalysts and environment modifiers. Overall, integrations of such organic architectures with inorganic and biological components resulted in innovative hybrids such as DSP, DSPC and COF systems. High performances have already been reached, especially toward photocatalytic H_2_ evolution (>60 mmol h^−1^ g^−1^), making these systems competitive with inorganic counterparts.

The possibility of molecular engineering has played a vital role in achieving these recent developments, with rational design enabling the integration of anchoring abilities, improved reactivity, intense and wide light absorption, high surface area, efficient charge separation and transport, and so forth. Synergistic combinations of such properties can in principle result in further enhanced catalytic activity. Nevertheless, the structure optimization of one parameter often collaterally impacts other properties, and deconvolution of individual effects remains challenging.

Despite these significant advances, many opportunities for further exploration persist. A better understanding of photocatalytic fuel mechanisms for polymers, COFs and other carbon nanoparticle‐based materials is desirable to reach better designs toward higher performance. There is scope for metal‐free electrocatalysts and the assembly of CO_2_‐reducing and full water‐splitting systems. A particular opportunity lies ahead in the exploration of redox transformations beyond classical solar fuels applications such as organic electro‐ and photoredox catalysis. The relatively unexplored possibilities offered by tuning the environment around the catalytic center and PS bears many promises and organic chemistry also allows for the development of nanoreactors to enable controlled catalysis in a confined environment.^56^ More robust and red‐light absorbing PSs^57^ are also in demand as well as a better understanding of aqueous media‐organic system interfaces and the development of oxidative chemistry.^58^ Finally, the integration of organic materials with biological systems,^59^ and the prospect of their high throughput analysis by robotics^60^ represent further exciting avenues of future research. We therefore envision many possibilities to employ organic chemistry in the future development of electro‐ and photocatalytic systems.

## Conflict of interest

The authors declare no conflict of interest.

## Biographical Information

*Julien Warnan is a Junior Group Leader at the Chair of Inorganic & Metal‐Organic Chemistry at the Technical University (TU) of Munich (Germany). He obtained a Ph.D. in organic chemistry in 2012 from the Université de Nantes (France). After postdoctoral tenures at King Abdullah University of Science and Technology (Saudi Arabia) and the University of Cambridge (UK), he joined TU Munich in 2019. His current research is focused on the development of organic chromophores, polymer systems, and hybrid materials toward solar‐driven fuel synthesis*.

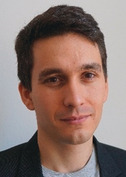

## Biographical Information

*Erwin Reisner is Professor of Energy and Sustainability at the University of Cambridge and a Fellow of St. John's College (UK). His laboratory explores the interface of chemical biology, synthetic chemistry, materials science, and engineering relevant to the development of solar‐driven processes for the sustainable synthesis of fuels and chemicals. He also chairs the Cambridge Creative Circular Plastics Centre (CirPlas) and the UK Solar Fuels Network (SFN), which coordinates the national activities in artificial photosynthesis*.